# Preventing chick culling in the poultry industry with a new biomarker for rapid in ovo gender screening

**DOI:** 10.1038/s41598-026-42524-w

**Published:** 2026-03-04

**Authors:** Nicolas Drouin, Hyung Lim Elfrink, Wouter Bruins, Slavik Koval, Chang Liu, David M. Cox, J. Bryce Young, Serge Desmoulins, Kelly Hoogkamer, Leonard van Bommel, Farzana Azam, James Wighton, Thomas R. Covey, Wil Stutterheim, Amy C. Harms, Thomas Hankemeier

**Affiliations:** 1 Metabolomics and Analytics Centre, Leiden Academic Centre for Drug Research, Leiden University, Einsteinweg 55, 2333 CC Leiden, The Netherlands; 2 SCIEX, 71 Four Valley Dr, Concord, ON L4K 4V8 Canada; 3 In Ovo, Haagse Schouwweg 12, 2332 KG Leiden, The Netherlands; 4 Agilent Technologies, 3 Avenue du Canada, 91940 Les Ulis, France

**Keywords:** Mass spectrometry, Metabolomics, Biomarker discovery, Gender screening, Animal welfare, Poultry industry, Predictive markers, Mass spectrometry, Metabolomics

## Abstract

Chicken eggs are one of the most consumed foods worldwide. However, the practice of chicken culling in the poultry industry involves unnecessary animal suffering and finding a way to put an end to this has become a societal priority. One approach that has been propagated as acceptable is based on the selection of female eggs early in the incubation process and the devitalization of the male eggs. It is with this objective in mind that we searched for a biomarker for early gender screening in eggs. Applying an untargeted mass spectrometry approach, we profiled allantoic fluid of different day-old eggs and identified the feature 3-[(2-aminoethyl)sulfanyl]butanoic acid (ASBA) as a strong biomarker for in-ovo gender prediction for day-9 old embryos. In the present work, we describe the identification of ASBA as a new biomarker in allantoic fluid for gender screening and the optimization of a high throughput assay using acoustic droplet ejection-mass spectrometry (ADE-MS). Special attention is given to the optimization of ADE-MS compatible liquid handling and the development of the data processing to ensure a reliable gender prediction. We have been able to accurately determine the gender of day-9 eggs in a cohort of 154 samples with a prediction accuracy of 95.5%, with a throughput of 1800 samples per hour for the prototype, which may vary in production systems.

## Introduction

Chicken eggs are one of the most popular cooking ingredients in every country over the world. Every year, more than 1.3 trillion eggs are produced by 4.7–6.5 billion of laying hens, representing a tremendous 80.1 million metric tons of eggs^1^. The productivity of laying hens drops anywhere between 1 and 3 years of age after which they are slaughtered and replaced. This causes a perpetual need for massive laying hen production. Unlike broiler chicken breeds, males from these egg-laying breeds do not produce enough meat to have any significant value in the chicken meat industry. Hatching males increases the costs in the laying hen industry. Furthermore, this poses an ethical problem: the need to cull the males. Currently, due to lack of viable alternatives, chick sexing is manually performed after hatching and, depending on the breed, is based on different colors of the down, or observation of the cloaca. While female chicks are kept for egg production, males are killed by mechanical methods such as maceration or asphyxiation^2,3^.

In the past decade, the concern for animal welfare has been growing and many countries are now legislating the practice of culling of male chicks, which results in the killing of more than 3.2 billion day-old male chickens every year^4,5^. Consequently, identifying economically viable alternatives to this practice has become a matter of considerable urgency. The *in ovo* gender determination is one of the most promising solutions. Since it takes 11 days for chick embryos to have a functional brain, it is possible to painlessly discard the eggs before this date^6^.

On average, a hatchery incubates up to 20,000,000 eggs per year. This means, on a 10-h work schedule basis, 200 days a year, 10,000 eggs are currently processed in this facility every hour. To be routinely integrated into a hatchery workflow, any set-up for gender prediction must comply with this throughput. In this feasibility study we aimed for a throughput of 1800 eggs per hour on a single system. To be viable, the technology cost per samples must remain low and the gender determination must ideally be as accurate as a manual gender determination post hatching (i.e. > 99%).

As any gendered species, egg gender can be easily determined with absolute certainty by genomics. Using this principle, He et al. developed simple, sensitive and robust PCR and qPCR approaches^7^. However, PCR based approaches are relatively expensive and slow. Some alternative methods have already been developed for this purpose, most of them based on optical technologies. In 2023, Kayadan and Uzun reported a significant correlation (r = 0.78) was observed between chick sex and Shape Index^8^. In 2011, Steiner et al. developed an FT-IR spectroscopy method to detect the chromosomic difference between males and females from the germinal disc^9^. However, this collection methods of cells presents high risks of viability for the embryo. More recently, the same group developed non-invasive Raman and fluorescence spectroscopy methods of the blood vessels located under the shell^10,11^. The combination of both spectroscopic measurements attained an overall prediction accuracy of 93%^12^. To date, in addition to the approach presented in this manuscript, three solutions are already commercialized. The first one, developed by Agri Advanced Technologies, is a spectrophotometric technique based on color analysis of the first feathers of chicks which are white for males and brown for females. Although non-invasive, fast and with a prediction accuracy of 97%, this method can only be employed from day-13 of brown chicken breeds^13^, causing ethical issues^6^. A method with similar ethical issues has been commercialized by Orbem using MRI-based technology for in-ovo sexing poultry eggs on day 12^14^. The third approach has been developed by Weissmann et al. and is now commercialized by Seleggt (Germany). In 2013, they identified estrone sulfate, a metabolism product of sex steroids, as a biomarker for in ovo early gender prediction and they developed an ELISA test for its detection^15^. However, immunoassays are expensive due to the development of selective antibodies and their production. Lastly, Plantegg developed and deployed an in ovo sexing method based on PCR^16^.

Untargeted LC–MS is a powerful strategy for biomarker discovery due to its sensitivity, capacity for high-throughput analysis, ability to identify and quantify complex biomolecules, and its potential to provide structural insights into biomarkers. After initial biomarker discovery, additional work needs to convert the biomarker detection method into a validated assay by improving quantification performances, or throughput for a clinical application, or in our case an industrial application requiring a minimum throughput of 1800 eggs per hour. To reach such an analysis speed, only direct MS introduction such as flow injection analysis (FIA), desorption electrospray ionization (DESI), laser diode thermal desorption (LDTD) or matrix-assisted laser desorption/ionization MALDI can be considered.

Accoustic mist ionization-mass spectrometry (AMI-MS) is the combination of an acoustic droplet ejection (ADE) system and a high voltage power supply to generate an ionized mist of droplets and an active transfer of the droplet(s) to a mass spectrometer (MS) using a field gradient. Using this set-up, it is possible to inject close to 3 samples per second without carryover or cross-contamination^17,18^. However, this technology lacks stability and robustness regarding the generation of the mist. For these reasons, an alternative, involving an open port probe interface (OPI)^19,20^ has been developed by SCIEX and is now known as ADE-MS and recently commercialized as Echo® MS. In this method of coupling the ADE to an electrospray source, the droplets are transferred to the ionization source through a continuous flow of solvent. This system can reach a throughput of more than 6 samples per second^21^.

The present study reports the discovery and characterization of a new biomarker using mass spectrometry-based analytical tools. The identification of the relevant features was first made using untargeted RPLC-MS analysis and subsequent structure elucidation of these features was achieved by the combination of several analytical approaches using MS/MS. We also describe a high throughput quantification method using ADE-MS technology for the quantification of ASBA in allantoic fluid from day-9 eggs for *in ovo* gender screening. We especially focused on the optimization of ADE-OPI-MS conditions and automated sample preparation compatibility. Using the optimized workflow, we were able to predict the gender of chick embryos with an accuracy of 95.5% and an analysis speed above 1800 samples/hour.

## Materials and methods

### Reagents

S-Propyl-L-Cysteine and 3-[(2-aminoethyl)sulfanyl]butanoic acid (ASBA) and the deuterated version, ASBA-d_4_, were synthesized upon request by Enamine (Kiev, Ukraine). S-(2-carboxypropyl)-Cysteamine was synthetized by the group of Prof. Byeong-Seon Jeong (Gyeongsan, South Korea). MS grade water was produced by a milliQ water generator. Formic acid (FA) and methanol were purchased from Acros Organics (Geel, Belgium) and Actu-All Chemicals (Oss, the Netherlands) respectively.

### Samples

Eggs used in this study came from brown chickens (VB1636 Brown Nick) and white chickens (Hy-Line CV 24) (Pluriton Netherlands b.v., Afferden, NL). Depending on the development stage, from 100 µL to 1 ml of allantoic fluid has been manually collected by carefully drilling a small hole and taking a sample through the air cell. A 0.22 Gauge needle on a syringe was used to extract the allantoic fluid without inflicting harm to the developing embryo. After collection, the allantoic fluid samples were snap frozen in liquid nitrogen before transport and were stored at − 80 °C prior to analysis.

During this study, 3 different sample cohorts were used: (i) the discovery cohort, (ii) the breed cohort, and (iii) the high throughput cohort. The first discovery cohort consisted of 50 Brown Nick eggs collected at day-7 and 60 eggs at day-8,9,10 and 11 after the start of the incubation. The breed cohort consisted of a total of 146 Brown Nick and 151 Hy-Line eggs, both collected at day-9, and these were used to assess the prediction power of the candidate biomarker in different chicken breeds.

The high throughput method was tested using allantois samples manually collected by puncture with an insulin syringe, at day-9 from 154 brown chickens (VB1636 Brown Nick). Approximately 1 mL of allantoic fluid was collected from each egg. After collection, samples were snap frozen in liquid nitrogen. After their transport from the hatchery to the laboratory, the allantoic samples have been stored at − 80 °C prior to analysis.

### Genetic gender determination by PCR

To determine the gender of the embryo at the early stages of its development, a PCR analysis was made using the primer 1237L and 1237H^22^. To do so, the embryo toes of each corresponding allantoic fluid sample have been collected and snap frozen in liquid nitrogen. The samples were then subjected to PCR analysis.

### Sample preparation for biomarker discovery

Allantoic samples from the discovery and breed cohort have been deproteinized with methanol. Briefly, 10 µL of the sample has been mixed with 90 µL of cold methanol and then vortexed. After centrifugation at 10,000 rpm at 10 °C for 10 min, the supernatant was collected. An equal fraction of each supernatant has been collected for each sample and then mixed to prepare QC samples which were analyzed at a regular interval between samples.

Prior to targeted analysis, samples from the validation study were spiked with a deuterated internal standard. Briefly, 100 µL of allantoic fluid has been mixed with 100 µL of a solution made of the deuterated internal standard at a concentration of 600 ng/mL in water, using a Tecan Freedom Evo liquid handling platform (Männedorf, Switzerland). QC samples have been prepared by mixing an equal volume of each raw samples and processed in parallel to individual allantoic fluid samples.

### RPLC-MS/MSMS

Untargeted metabolite analyses were performed on an ACQUITY UPLC from Waters (Milford, USA) directly coupled to the ESI jet stream nebulizer of an Agilent 6530 series ESI-Q-TOF high-resolution mass spectrometer from Agilent Technologies (Waldbronn, Germany). The separation was performed with a Waters AccQ-Tag Ultra column (1.7 μm, 2.1 mm × 100 mm), thermostated at 60 °C. The LC gradient has been described previously in the analytical chromatography section of our previous work^23^ but with 2% formic acid added to both mobile phases. MS acquisitions were done in ESI positive mode using full scan mode with a mass range from 100 to 1000 m/z and used a lock mass for improved mass accuracy. MS/MS data were recorded at 5 and 25 eV using an inclusion list of targeted features.

### HILIC-MS

HILIC-MS analyses were performed using an Agilent 1260 Infinity LC system directly coupled to a DuoSpray™ ionization source of a Sciex 6600 TripleTOF (Darmstad, Germany). The separation was performed with a Zic-cHILIC column (3 μm, 2.1 mm × 100 mm, 100 Å), purchased from Merck Millipore and thermostated to 25 °C. The gradient is similar to published HILIC chromatography^23^ but used 5 mM of ammonium acetate instead of ammonium formate and a flow rate of 0.2 ml/min. MS acquisitions were performed in ESI positive mode using full-scan acquisition mode with a mass range from 50 to 1500 m/z.

### High throughput sample preparation

A pooled sample for method optimization has been prepared by merging an equal volume of allantoic fluid coming from 150 random eggs. To mimic a routine situation, a preliminary prototype of an automatic egg sampler was used to punch the eggs and collect allantoic fluid from each egg in a 96 well plate. During the first iterations of this prototype, the sampling success rate was about 60%, meaning 40% of the wells were empty or insufficiently filled with sample. Sample preparation was performed using an Agilent Bravo liquid handling platform. In the same tips, 15 µL of a solution containing 600 ng/mL of IS in 2% FA was aspirated, directly followed by 15 µL of sample. Tip content was dispensed into an ADE compatible 384 well plate and mixed by 10 iterations of gentle aspiration and dispense. Then 10 µL was aspirated. After the dispense of the excess of 10 µL to waste, the tips were washed using a solution of ethanol 70% and dried by slowly blowing 200 µL of air.

### ADE-MS

**Fig. 1:**
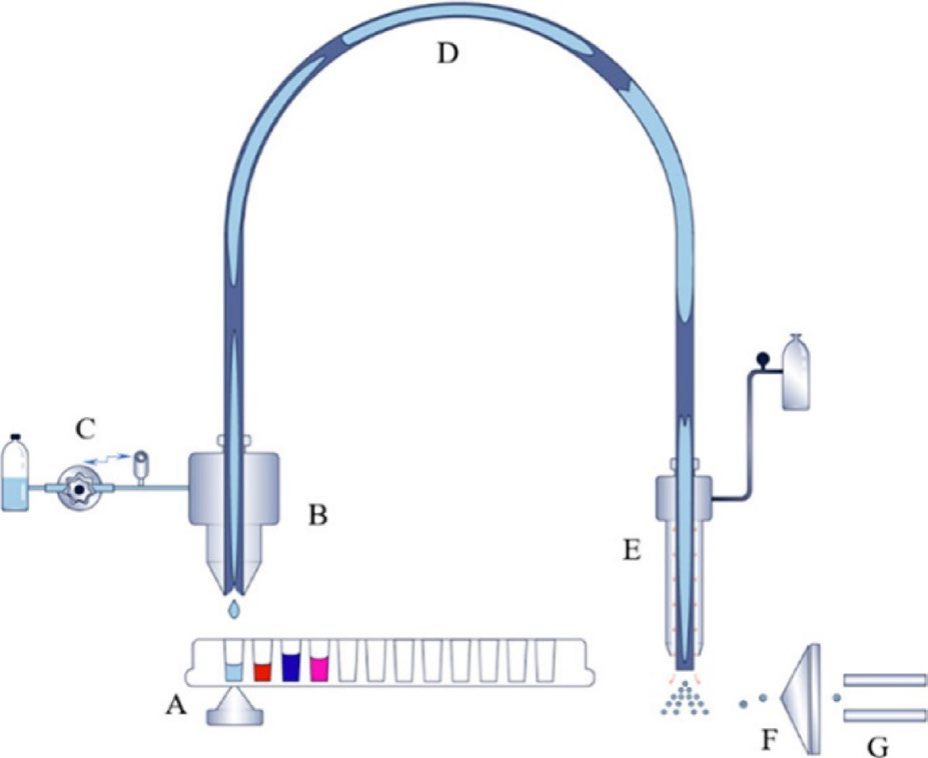
Diagram of the research breadboard. (A) Acoustic transducer dispensing sample from microtiter plate wells; plate moves on an x–y stage. (B) Open port probe interface. (C) Make-up pump delivering the carrier solvent to the OPI at 200 μL/min. (D) A 68 cm OPI fluid transport tube. (E) Venturi pump/ESI nebulizer drawing the transport fluid from (B). (F) Atmosphere to vacuum aperture. (G) Mass spectrometer. Reproduced with permission from Liu et al.^19^.

The ADE-OPI-MS system used during this study was a prototype based on an acoustic liquid handling comprising a modified EDB Biosystem Gen4 + where the rotary function of the receiver plate has been disabled and a proprietary open port probe interface mounted at 2 mm from the source plate (Fig. 1). The OPI was centered over the acoustic transducer central axis within ± 100 μm using the ATS-100 software and 5 nL droplets were generated using the water calibration and a pause of 1.2 s was observed between injection of each sample.

An LC 1260 series from Agilent (Waldbronn, Germany) has been used to deliver the make-up solvent at 200 µL/min. A fused silica capillary of 160 cm and 50 μm ID, purchased from BGB Analytik (Harderwijk, The Netherlands), was used to generate 200 bar backpressure.

The ADE-MS system was hyphenated to a 6500 + QTrap (SCIEX, Concord, ON, Canada), equipped with an Optiflow source. MS experiments were performed in the positive ionization mode using the selected reaction monitoring (SRM) acquisition mode. The precursor and product ions for ASBA was 164.1 to > 147.0 and ASBA-d_4_ was 168.1 to > 151.0, both with collision energy 14 V and dwell time 40 ms. The solvent transfer tube was a PEEKsil, having dimension of 800 μm OD × 250 μm ID × 60 cm length, and was vertically aligned in the center of the OPI. It connects with a 250 μm ID union to a modified stainless-steel electrode/probe assembly. The ion source gas 1 and 2 pressures were fixed at 90 and 85 psi, respectively, with a temperature of 300 °C for both. The ion spray voltage, declustering potential and collision cell exit potential were adjusted to 4000 V, 80 V and 10 V, respectively. The curtain and collision gas were set at 25 psi and “medium”, respectively.

Data acquisition and instrument control were monitored using SCIEX Analyst version 1.6.2 (SCIEX, Concord ON, Canada).

### Data processing

The raw data from both discovery and breed cohorts were pre-processed using Agilent MassHunter Workstation Software Profinder (Agilent, Version B.06.00, Build 6.0.606.0. The Batch Recursive Feature Extraction was performed on the raw files loaded into the software. The following settings were used, settings not mentioned were as used as default by the software. Molecular feature extraction was performed for peaks between 0 and 8 min, for m/z between 100 and 900 m/z and with a minimum count of 300. Peak binning and RT alignment have been done using a RT window of 1% ± 0.15 min and a mass window = 5.00 ppm ± 2 mDa. The maximum number of features was set to 2000. Recursive feature extraction was made using Find by Ion mode, using matching tolerance of ± 1.5 min for retention time and 35 ppm of symmetric error on the m/z. The peak area extraction of the features was done using Agile algorithm. Duplicate features, artefact features from the data preprocessing, and features present in less than 90% of the samples were manually removed, as well as samples with less than 80% of the total amount of features present.

The raw data from the validation cohort has been processed using Skyline^24^. Finally, the statistical treatment of the extracted features has been done using MetaboAnalyst 5.0^25^ and R packages.

### ADE-MS data processing

ADE-MS technology allows acquisition at a very high frequency. Therefore, it is impossible to directly obtain a data file for each sample and instead, the data from all the samples in a plate are present in the same raw data file. For the processing of data acquired with this prototype, it is necessary to split the file to generate individual results for each sample. This splitting step was possible thanks to custom software developed by SCIEX. Briefly, the peak corresponding to the injection of the first sample was detected using a threshold based on the intensity of the most intense peak present. Then, the ADE report containing the time interval between two consecutive ejection events (i.e. two consecutive samples) was used to assign each data point of the chromatogram to a specific sample and finally to split the raw data into individual files. In this study, we chose to use 10% of the maximum peak intensity.

## Results

### Allantoic fluid is an accessible and non-damaging fluid for biomarker discovery and routine analysis

The allantois is a hollow-sac structure developed by the embryos and has two main functions. As it is highly vascularized, the allantois aids in gas exchange of the embryo, supplying oxygen for the red blood cells that passed through the eggshell. Its second function is to collect the nitrogenous waste produced by the fetus, thus making the composition of the allantoic fluid close to mammalian urine. It is also interesting to note that having the lowest fluid density, the allantoic fluid is easily accessible after puncturing the shell through the air sac. Consequently, the collection of a reasonable volume, dependent on the development of the fetus, of this biofluid is not detrimental to the fetus and makes it a good source for potential biomarkers^15^.

### Untargeted metabolomics approach for the discovery of gender specific biomarkers in allantoic fluid

**Fig. 2:**
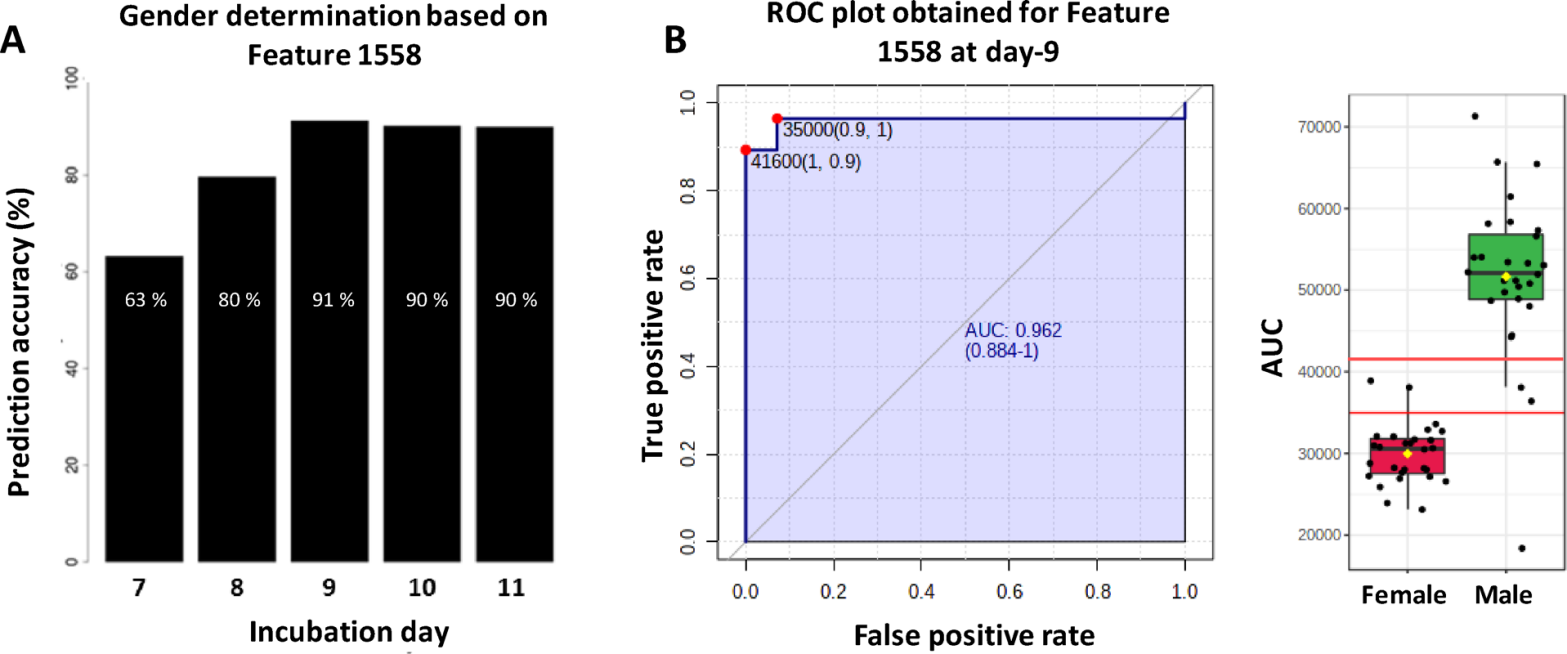
(**A**) Logistic regression classification model on single feature. We evaluated single predictor models for all measured features and observed the best accuracy using Feature 1558 (F1558), which provides > 90% accuracy at day-9. (**B**) ROC plot of F1558 at day-9 and its associated boxplot.

Metabolomics based biomarker discovery methodologies rely on the detection of a very large variety of compounds in order to maximize the chance of finding interesting hits^26,27^. For this reason, an untargeted liquid chromatography with mass spectrometry (LC–MS) approach was chosen using positive electrospray mode on a high-resolution Q-TOF. The discovery cohort comprised 50 eggs for day 7 and 60 at days 8 to 11. In total, 1954 peaks with unique retention time and mass, so called features, were detected in the LC–MS chromatograms with this method. After manual curation of the data, 1390 features were considered, and the gender was modeled as a binary outcome with sampling day and measured features as model predicator. The model was further optimized using backward elimination techniques^28^, validated using cross-validation^29^ and evaluated by the percentage of correctly predicted genders, male and female combined. This approach identified a particular feature (F1558) as the most promising biomarker candidate with a prediction rate up to 91% at day-9 (Fig. 2A).

This particular feature presented similar prediction power for days 9 to 11. However, during chicken embryonic development, the neural tube needs approximately 50% of the 21 days of the total gestation duration to turn into a functional brain^6,30–32^. This indicates that chicken embryos can be euthanized up to 11 days after laying, before they can perceive pain. In addition, we found the collection of allantoic fluid from an individual egg during day-9 of the development did not lead to a loss of hatchability of the embryos. For ethical reasons, we choose to focus on day-9 and disregarded later days. A univariate receiver operating characteristic (ROC) analysis for F1558 (shown in Fig. 2B) shows a sensitivity of 96.4% (IC_95%_ = 89.3–100%) and a specificity of 92.9% (IC_95%_ = 85.7–100%) using a peak area threshold of 35,000 counts.

**Fig. 3:**
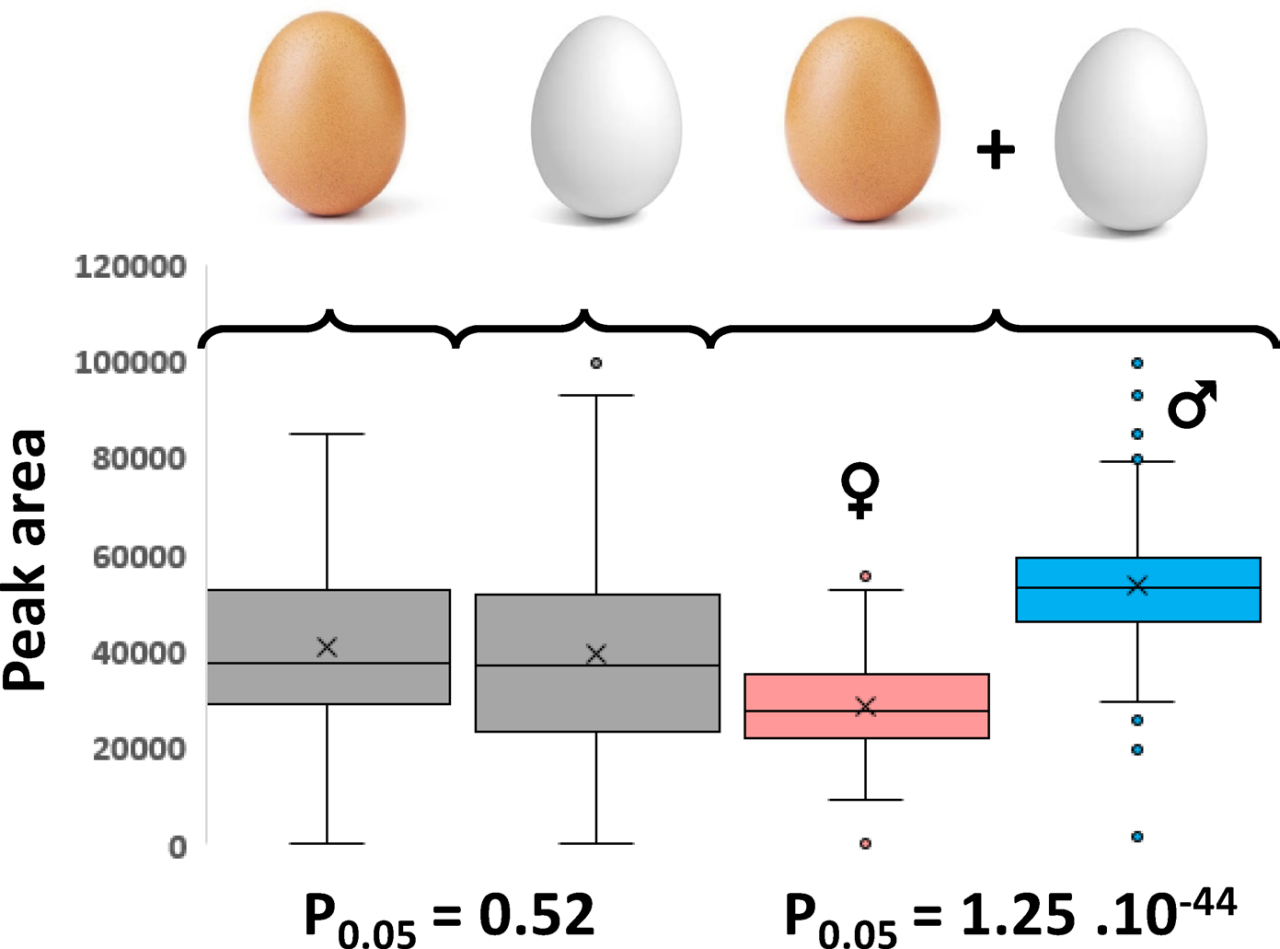
Box-plot comparison of the white and brown eggs populations.

The first experiment identified a potential biomarker of gender from day-8 to 11 in H&N Brown Nick chickens. To evaluate the prediction capacity of this feature in different chicken lines, a new experiment was performed with the analysis of a breed-cohort of allantoic fluid coming from Brown Nick chickens and white Hy-Line chickens (146 and 151 samples respectively) collected at day-9. As shown in Fig. 3, we did not find any statistical difference of the average biomarker level in the overall population of each chicken line (p_0.05_ = 0.52). When considering brown and white eggs together, we find the endogenous level of the biomarker was significantly different between male and female eggs (p_0.05_ = 1.25 × 10^−44^). It has thus been possible to set a common intensity threshold, independent of the chicken lines, giving a prediction accuracy above 89.5%.

These results clearly highlight the strength of the discovered feature for early gender determination of chicken embryos 9 days after the egg-laying regardless of chicken species.

### 3-[(2-Aminoethyl)sulfanyl]butanoic acid was identified as a gender-specific biomarker

Unambiguous identification of the biomarker requires the comparison of the feature of interest to reference compounds using different characterization approaches to obtain orthogonal information. Therefore, for confident annotation of an unknown feature, at least 2 independent parameters need to match the reference compound, such as liquid chromatography retention time, accurate mass or mass spectrometry fragmentation pattern (MSMS spectra)^33,34^.

**Fig. 4:**
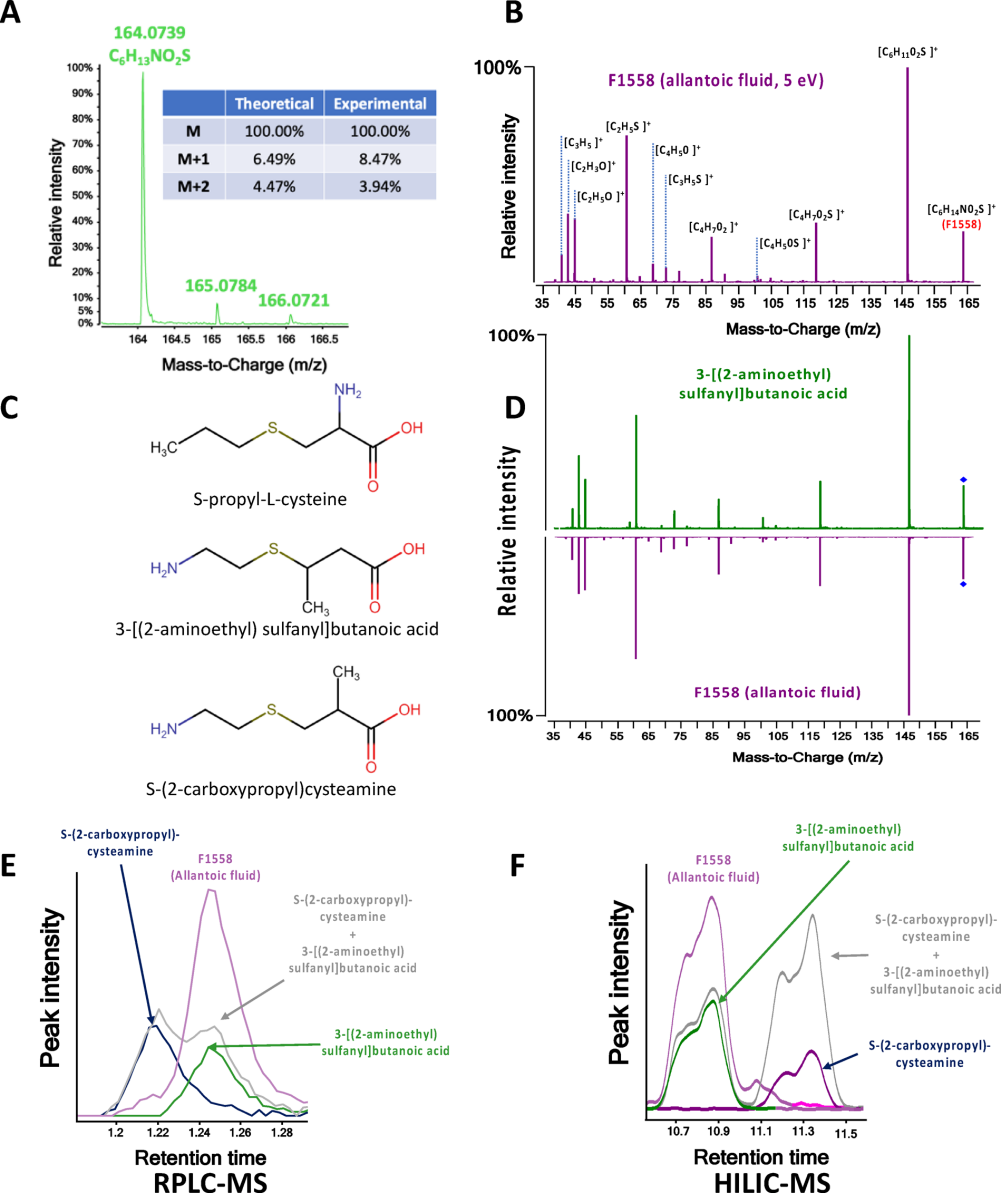
Identification process of F1558 (**A**) determination of the elemental composition, (**B**) MSMS spectra of F1558 and elemental composition of fragments, (**C**) possible structures, (**D**) comparison of fragmentation pattern of F1558 and 3-[(2-aminoethyl)sulfanyl] butanoic acid, (**E**, **F**) retention profile of F1558, S-propyl-L-cysteine, S-(2-carboxypropyl)-cysteamine and 3-[(2-aminoethyl)sulfanyl] butanoic acid in RPLC and HILIC mode respectively.

The accurate mass of the protonated form of F1558 has been accurately measured as m/z 164.0739. This mass was assigned by Agilent MassHunter as corresponding to the chemical formula C_6_H_13_NO_2_S which was confirmed by the isotopic distribution showing the specific pattern of sulfur atoms (Fig. 4A). In order to identify the biomarker, we undertook the identification of its MSMS fragments obtained after RPLC-MSMS of a pooled allantoic sample (Fig. 4B). Based on the fragmentation pattern, we proposed different structural isomers, whereby S-propyl-L-cysteine, S-(2-carboxypropyl)-cysteamine, and 3-[(2-aminoethyl)sulfanyl] butanoic acid were the most likely to produce the same fragments (Fig. 4C). Since they were not commercially available, these compounds were synthesized on our request.

The fragmentation pattern of the biomarker F1558 was then compared to the MSMS spectra of the three candidate compounds. As shown in Fig. 4D, 3-[(2-aminoethyl)sulfanyl] butanoic acid shares all its fragments with F1558 whereas the two other compounds only had 2. Additional confirmation of the identity was obtained by comparing retention time profiles in two orthogonal chromatography methods. After injection in RPLC mode, S-propyl-cysteine was discarded as its retention time (RT) differed by more than 1 min from F1558 (data not shown). The RT obtained for 3-[(2-aminoethyl)sulfanyl] butanoic acid and S-(2-carboxypropyl)-cysteamine were slightly different, and two peaks were clearly observed when co-injected (Fig. 4E). We found the RT of 3-[(2-aminoethyl)sulfanyl] butanoic acid was the same as F1558. Second, we undertook a HILIC comparison. As shown in Fig. 4F, in this mode, 3-[(2-aminoethyl)sulfanyl] butanoic acid and S-(2-carboxypropyl)-cysteamine gave two baseline separated peaks. The peak shape and the RT of 3-[(2-aminoethyl)sulfanyl] butanoic acid were strictly identical to F1558. Therefore, combining all these orthogonal data together, we were able to unequivocally identify 3-[(2-aminoethyl)sulfanyl]butanoic acid (ASBA) as being the feature of interest F1558.

### High throughput screening

To be easily implemented and economically viable, the embryo gender assay should be able to measure at least 1800 samples per hour. Such throughput already represents a challenge, but it is enhanced by the requirement of the sexing accuracy to reach a minimal gender prediction accuracy of 95%. Female embryos presented a lower concentration of ASBA than male. However, due to the biological and analytical variations, we found the highest female and the lowest male concentrations are separated by a concentration difference less than 10% and are sometimes mixed.

**Fig. 5:**
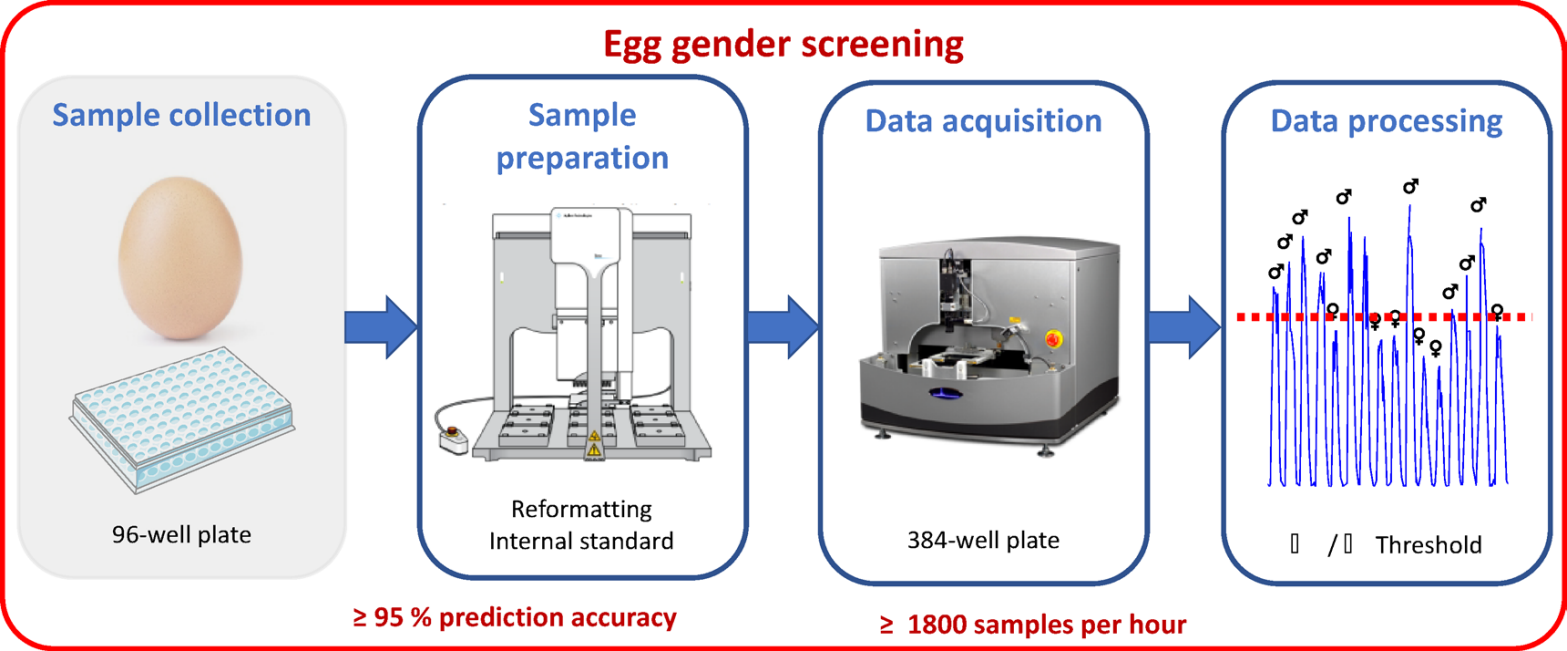
Workflow and requirements of the industrial egg sexing assay.

To reach such throughput and prediction accuracy, several steps must be optimized, from the sample collection to the model predicting gender (Fig. 5). The high throughput sample collection relies on engineering a high throughput sample collector able to punch the egg shell and collect the sample without impacting the further development of the embryo. In this study, we focused on the development of high throughput sample preparation and data acquisition method using the Agilent Bravo with a 96-tip head and a prototype of ADE-MS platform. In addition, we have developed a processing method to interpret the data and predict the gender of the embryos.

### ADE-MS optimization

The ADE-MS system is the combination of an acoustic transducer and an open port probe interface (Fig. 1). Acoustic droplet dispensing requires that the depth of the liquid is measured for the focal point of the sound waves to be set to irradiate the correct area of the liquid surface and achieve the targeted volume. To do this, the acoustic transducer measures the liquid level in the well by pulsing an acoustic wave at the bottom of the sample well through a coupling liquid (i.e. water) and measuring the interval of time between the reflected signal of the well-sample interface and the meniscus of the sample^19^. After its ejection, the droplet flies in a vertical motion and is captured by the OPI into a stream flow of solvent and is diluted and carried to the MS through the transfer capillary.

Droplets can be ejected at a frequency of 800 Hz. Consequently, the main limitation of the throughput appears to be the transfer speed of the droplet to the MS which is limited by the flow rate of the carrier solvent. Because the OPI is an open interface, the rate of the carrier solvent through the transfer capillary is dependent on the back pressure generated by the capillary and Venturi effect produced by the gas flow rate of the pneumatically assisted ESI source and is also dependent on the viscosity of the solvent. In order to maximize the flow rate and the throughput, the ADE-MS was equipped with a transfer capillary made of PEEKsil with a length of 60 cm, an inner diameter of 200 μm and modified with an 8 cm long and 250 μm ID stainless steel ESI probe assembly.

**Fig. 6:**
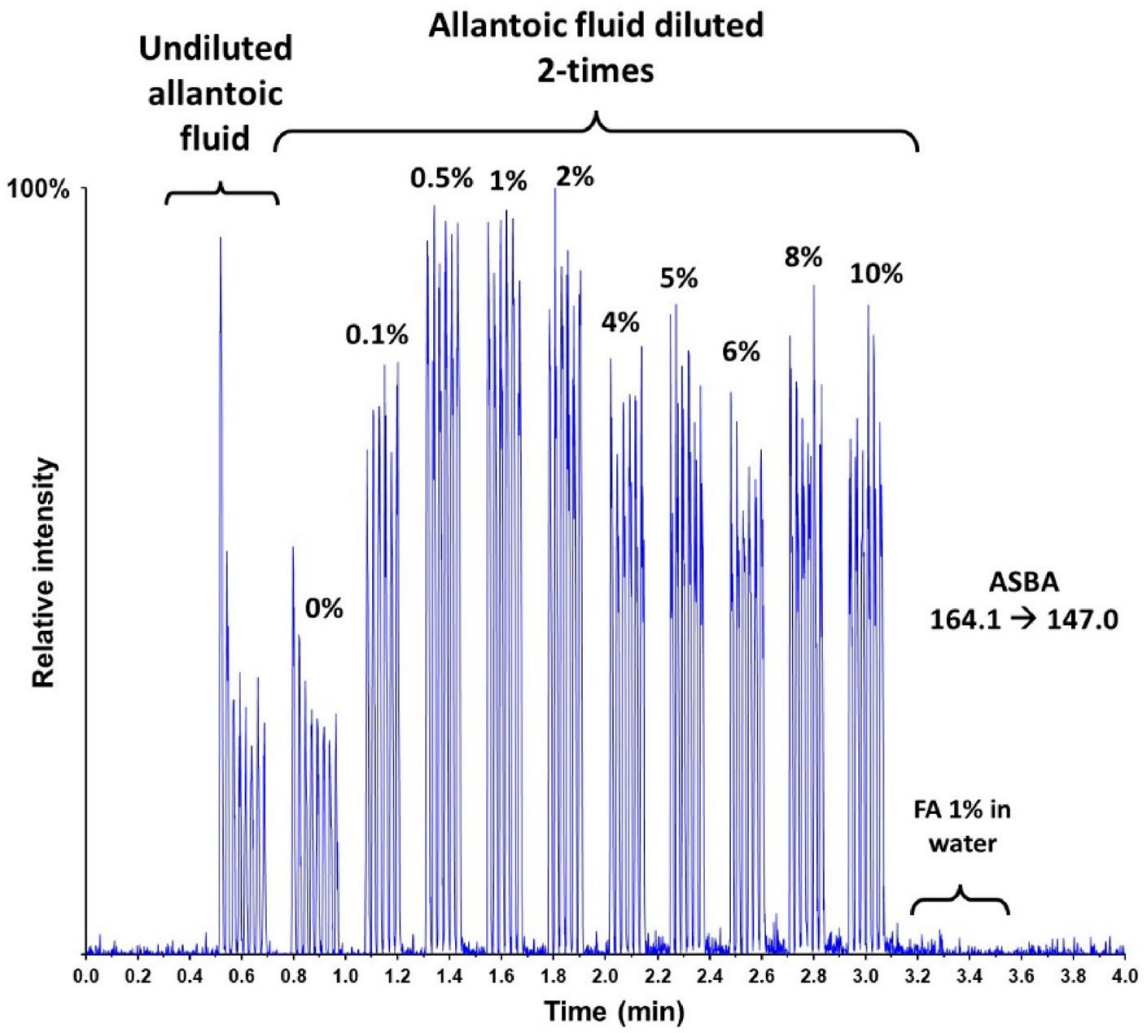
Influence of the sample dilution and the amount of formic acid spiked. Percentages correspond to the final concentration of formic acid.

In order to increase the sensitivity of the method, we investigated different sample compositions. As shown in Fig. 6, raw samples and samples diluted 2-times with pure water give approximately the same signal for the endogenous level of ASBA. For method robustness purposes, we decided to proceed with a 2-times dilution of the allantoic fluid with water containing 2% of formic acid, for a final FA concentration of 1%.

Since ASBA is an endogenous compound and no blank matrix is available, the limit of detection (LOD) and quantification (LOQ) have been estimated by injection of increasing concentrations of ASBA-d_4_ in a pooled allantoic sample, assuming ASBA and ASBA-d_4_ have the same MS response. Therefore, we determined the LOD (S/N > 3) was 5 ng/mL while the LOQ (S/N > 10) was between 10 and 20 ng/mL in 15 uL allantoic fluid starting material which is an acceptable sensitivity considering the threshold concentration between male and female embryos is in the range of 100 ng/mL.

### Sample preparation optimization

The robustness of the overall method needs to meet requirements of an industrial process, so we chose to use ASBA-d_4_ as a deuterated internal standard (IS). With this approach, it is possible to compensate for intra batch variation of the matrix effect, as well as injection variability and long-term source fouling.

**Fig. 7:**
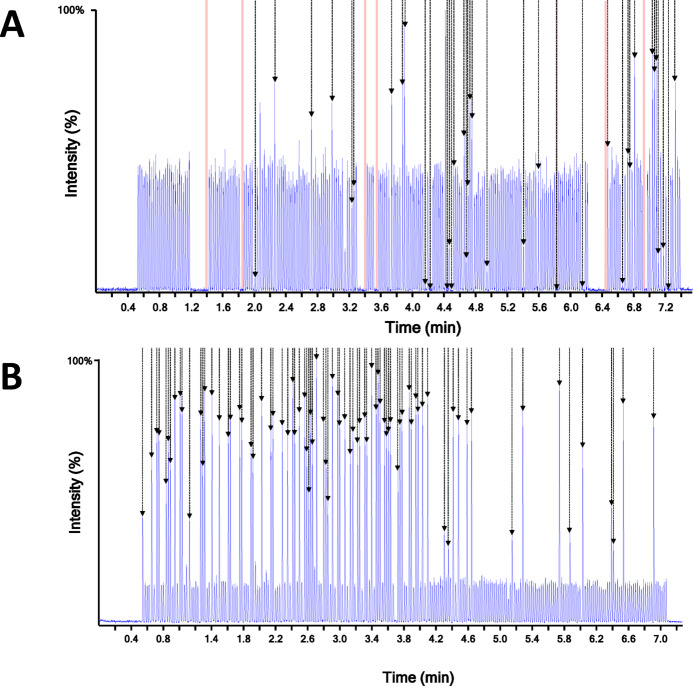
Optimization of the sample loading procedure. (**A**) and (**B**) represent the default and the optimized parameters for the liquid transfer, respectively. In both cases, the transition corresponding to ASBA-d_4_ is displayed. The black arrows and the rose areas represent the samples not or only partially collected by the robotic egg sampler and the samples presenting an ejection error respectively. Samples have been injected with a pause of 1.8 s between 2 consecutive samples.

With conventional LC–MS approaches, the data acquisition is the limiting factor to the throughput. However, ADE can measure a sample every 2 s, shifting the bottleneck to the sample preparation step. Therefore, to maintain a steady flow of samples without a traffic jam, the sample preparation cannot be longer than 2 min for a 96-well plate per sample preparation module. However, in return for a very high frequency analysis, ADE-MS is more prone to injection error than a classical injector using a syringe. Therefore, to maintain a high prediction accuracy, it is important to have robust sample volume transfer for stable ejection of samples. Consequently, we evaluated and optimized the robotic sample preparation and the plate loading steps based on samples collected by the first iteration of a homemade automatic egg sampler. This robot was able to collect allantoic fluid from an egg directly into a 96 well plate. In its first iteration, many factors lead to failed sampling, such as a clogged syringe or incorrect egg positioning, resulting in only 60% of the eggs correctly sampled and 40% of the wells empty or under sampled, but this was good enough to evaluate the ADE performance. As shown in Fig. 7A, after the aspiration in the same tip of the IS and allantoic fluid consecutively, their dispense to the 384-well plate and their mixing in the narrow well, ejection errors were reported for 7 samples. These injections errors were due to the presence of air bubbles preventing the acoustic ejector system from detecting the meniscus.

Next to this, we can observe in Fig. 7A that some samples don’t present any signal although they didn’t create an ejection error. These samples correspond to missing allantoic fluid samples in the 96-sample plate. Therefore, only the internal standard was spotted and not diluted. The low viscosity of the IS solution doesn’t allow the formation of air bubbles at the surface of the well, but on the other hand, the missing volume in the well makes the liquid transfer unsuitable for the ADE process.

As shown in Fig. 7B, no injection error was reported over 240 consecutive samples. In addition, the wells not containing allantoic fluid are distinguishable and gave an MS signal for the IS approximatively 4 times higher than mixed with the allantoic fluid. This observation is in accordance with the absence (or partial) of dilution of the IS (i.e. with the egg sample) and the absence of matrix effects. Consequently, the developed method ensured a signal for more than 99% of samples spotted into the analysis plate, with total sample pretreatment of 96 s, including the reuse of the tips after a cleaning step of the tips with water. This washing step was notably important to drastically decrease the cost of the analysis as well as the consumption of plastic.

### Carryover and process variability

Typically, carryover comes from the parts of the instruments in direct contact with different samples, usually from the injector. In the present workflow, although they are washed, reusing tips in the Bravo liquid handling platform used for sample preparation represents a potential source of carryover. In order to quantify the carryover, the same tips were used to process 60 identical pooled samples 3 times in the 3 firsts quadrants of the 384 well plate. Then 60 blank samples made of milliQ water were processed using the same tips to transfer samples to the 4th quadrant.

**Fig. 8:**
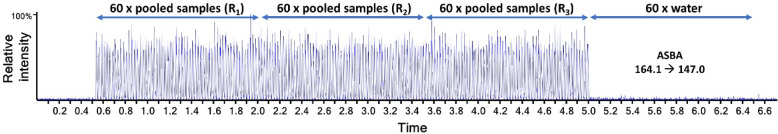
Carryover evaluation (R_1-3_: replicate 1 to 3). Samples were injected with a pause of 1.2 s between two consecutive injections.

As shown in Fig. 8, the absence of a signal from water samples shows the absence of carryover from the tips after being used for three consecutive sample preparations as well as the absence of carryover from the injection process.

To evaluate the process variability of the entire workflow (i.e. sample preparation and data acquisition), 60 wells of a 96-well plate were spotted with a pooled allantoic fluid sample and the plate was processed 4 times into the same 384-well plate. Over the 240 samples measured, the process variability presented a CV better than 6%. In addition, with a time delay of 1.2 s between 2 consecutive injections and including the instrumental delay of the system check inherent to every plate analysis, it is possible to analyze 240 samples in 6.5 min, so a throughput of 1.63 s/sample including both system checks and injection time.

### Threshold determination and prediction accuracy

To develop an economically viable gender screening test, a minimum of females has to be discarded. Although ASBA presents a high sensitivity for the prediction of gender, the determination of a cut-off value remains extremely difficult since preliminary data has shown the concentration difference of ASBA between male and female is in the 10% range.

In order to evaluate different threshold approaches, 154 samples, coming from two different batches of day-9 eggs, have been analyzed processed using the developed workflow.

**Fig. 9:**
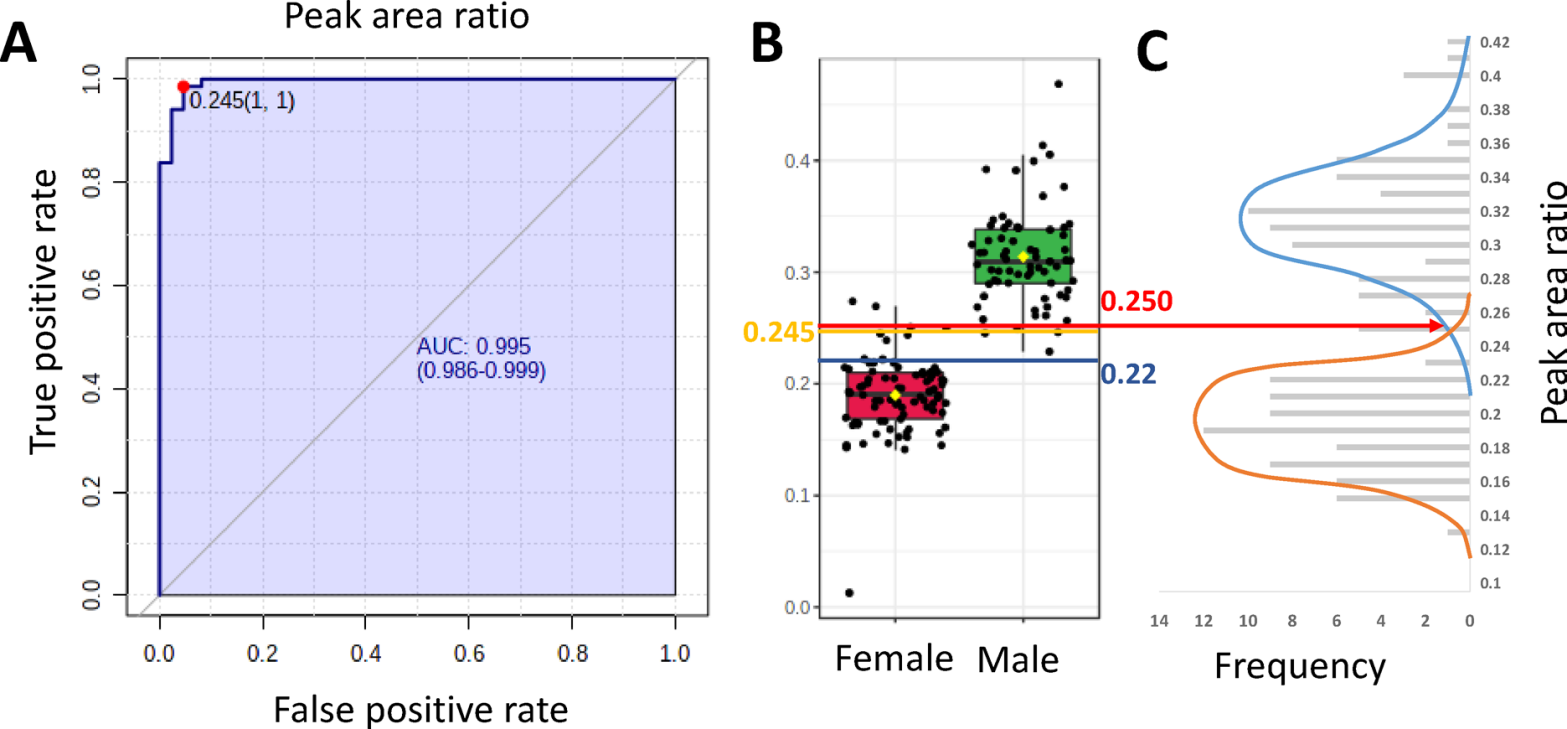
Performance of the method to predict the male gender of chick embryos at day-9. (**A**) ROC plot describing the male gender prediction performance of the ADE-MS method using ideal a posteri threshold. (**B**) Classification of the peak area ratio according to the true gender. The blue line represents the median of the population while the yellow and the red line represent the ideal and the dynamic threshold respectively. (**C**) Population frequency of the peak area. The orange and blue curves represent a simulation of the female and male distribution among the sample cohort.

As shown in Fig. 9A, the theoretical best threshold of peak area ratio for this particular batch was found to be 0.245, with a sensitivity of 98.5% (IC_95%_ = 94.1–100%) and a specificity of 94.2% (88.4–98.8%).

After manual inspection of the data, we find 0.251 as optimal cut-off value giving the overall best prediction rate, i.e. 95.9% for both batches. However, such retrospective threshold determination is not compatible with a routine application and an alternative threshold determination should be implemented. Indeed, the fixed threshold approach suffers from major drawbacks such as egg flock variation, IS batch preparation or changes in the IS concentration due to degradation or slow evaporation of the solvent. To overcome these issues, we decided to investigate a dynamic approach, using statistical parameters specific to each sample plate. We first evaluated the median ratio ASBA/IS (Fig. 9B). In this situation, a sensitivity of 100% and a specificity of 89.5% were found, resulting in an overall gender prediction accuracy of 94.6%.

The second approach we have evaluated is based on population distributions (Fig. 9C). With a sufficient sample cohort, it becomes possible to distinguish two Gaussian curves in the distributions of the population. As shown in Fig. 9C, the intercept of both curves can be used as the cut-off value. In the present case, although the sample cohort was limited, the dynamic cut-off value was found to be 0.250, which is close of the ideal threshold. Using this dynamic threshold, we ended up with 68 male eggs with 3 false predictions and 86 female eggs with 4 false predictions, giving a sensitivity of 96.5% (IC95% = 90.0–99.2%), specificity of 94.2% (IC_95%_ = 85.8–98.4%) and a global prediction accuracy of 95.5% (IC_95%_ = 90.9–98.2%). This approach is also less susceptible to population disequilibrium observed in small sample cohorts. In routine analysis, we can expect even higher prediction performance measuring a full plate of 384 samples.

## Discussion

With the changing mindset and the rapid evolution of legislation, finding alternatives to the culling of day-old male chicks has become a societal issue. In this context, developing a method to *in ovo* discriminate male and female chickens, before the full development of the central nervous system appears to be a humane solution to this problem. Applying untargeted LC–MS approaches on limited sample cohorts, we were able to identify a candidate biomarker feature, and with it able to accurately predict the gender of chicken embryos from day-8 to 11 after egg-laying. We have also demonstrated the prediction accuracy of this candidate biomarker across two different chicken breeds. After its identification, we have been able to confirm that 3-[(2-aminoethyl)sulfanyl]butanoic acid (ASBA) is a powerful biomarker for early gender screening.

An average sized hatchery has to process about 10,000 eggs per hour. To reach such a throughput and considering an ideal case of a total analysis time of 1 min per sample with a state-of-the-art LC–MS platform, 180 instruments would be necessary.

ADE-MS, thanks to its unique injection system, represents a valuable alternative for such a high throughput assay. Indeed, we have been able to circumvent some of the main problems encountered in LC–MS or flow injection analysis using conventional LC injectors. Because no part of the system is in contact with the sample, ADE-MS does not suffer from carryover problems from injection valve or needle contamination. This allows for an injection speed much higher than conventional LC injectors. In this regard, the current limitation of the system is only dependent on the transfer capillary, which limits the flow rate of the carrier solvent, and the speed of the ADE-MS which was limited to 1.5–2 s per sample for the prototype we used in this study. In order to come to a method which works smoothly with current hatchery processes, there is a clear need for robotization of sampling, lab automation and parallelisation. We believe 10.000 eggs per hour should be doable and by incorporating additional markers, 99% accuracy could possibly be realized. This could come at a cost of several millions of equipment, which is a steep investment, but would be used for 20.000.000 tests per year, per hatchery, with very limited cost of consumables and labor. In turn this would equate to only cents on the dollar per test done, which is low enough to be attractive for farmers.

The main inconvenience of the ADE-MS system lies in the ejection process. Each sample well has to be free of air bubbles. By fine-tuning the sample preparation methods, we were able to avoid laborious steps without compromising throughput and repeatability. Indeed, including a washing step of the tips to reduce the costs, the developed method was able to process a sample plate in 96 s (1 s per sample), with more than 99% of injection success rate.

Finally, we developed a dynamic threshold approach based on the ratio ASBA/ASBA-d_4_ to classify the eggs. This approach uses the distribution of the population to overcome many issues such as the inhomogeneous male–female population, the variability of the IS concentration due to evaporation or batch effect, or the day-to-day variation. In the present study, although this approach is more suitable for large batches of eggs, we were able to accurately predict with a global gender accuracy of 95.5%. Although already quite high, this is not on par with current manual sexing, which is at 99%. We believe additional biomarkers could possibly be utilized to increase accuracy.

Considering the sample preparation can be done in parallel to the data acquisition, the developed workflow can process more than 2000 samples per hour. We are convinced this new approach will open the door to new agro-food applications, with the possibility to screen the totality of a batch instead of a subset.

## Data Availability

The datasets generated and/or analysed during the current study are not publicly available due to potential infringements on an IP agreement but are available from the corresponding author on reasonable request.

## References

[1] WATTPoultry. Poultry trends 2019: The Statistical Reference for Poultry Executives: Watt Executive Guide to World Poultry Trends. https://www.wattagnet.com/egg/article/15534714/ (2019).

[2] EFSA Panel on Animal Health and Welfare (AHAW) et al. Killing for purposes other than slaughter: Poultry. *EFSA J.* **17**(11), e05850. 10.2903/j.efsa.2019.5850 (2019). 32626157 10.2903/j.efsa.2019.5850 PMC7008794

[3] European Union, Council Regulation (EC) No 1099/2009 of 24 September 2009 on the protection of animals at the time of killing. *Official Journal of the European Union.* Document 32009R1099. http://data.europa.eu/eli/reg/2009/1099/oj (2009).

[4] Blide, D., & Montel, S. Motion for a resolution, European Parliament, B8-0625/2015 (2015).

[5] United Egg Producers, United Egg Producers updated statement on male chicks, Retrieved from https://unitedegg.com/united-egg-producers-updated-statement-on-male-chicks-2/. Accessed 30 Jan 2026 (2021).

[6] Close, B. et al. Recommendations for euthanasia of experimental animals: Part 2. DGXT of the European Commission. *Lab. Anim.* **31**(1), 1–32. 10.1258/002367797780600297 (1997). 9121105 10.1258/002367797780600297

[7] He, L. et al. Simple, sensitive and robust chicken specific sexing assays, compliant with large scale analysis. *PLoS ONE* **14**(3), e0213033. 10.1371/journal.pone.0213033 (2019). 30822330 10.1371/journal.pone.0213033 PMC6396912

[8] Kayadan, M. & Uzun, Y. High accuracy gender determination using the egg shape index. *Sci. Rep.* **13**, 504. 10.1038/s41598-023-27772-4 (2023). 36627389 10.1038/s41598-023-27772-4 PMC9832119

[9] Steiner, G. et al. Gender determination of fertilized unincubated chicken eggs by infrared spectroscopic imaging. *Anal. Bioanal. Chem.* **400**(9), 2775–2782. 10.1007/s00216-011-4941-3 (2011). 21479544 10.1007/s00216-011-4941-3

[10] Galli, R. et al. In ovo sexing of domestic chicken eggs by Raman spectroscopy. *Anal. Chem.* **88**(17), 8657–8663. 10.1021/acs.analchem.6b01868 (2016). 27512829 10.1021/acs.analchem.6b01868

[11] Galli, R. et al. In ovo sexing of chicken eggs by fluorescence spectroscopy. *Anal. Bioanal. Chem.* **409**(5), 1185–1194. 10.1007/s00216-016-0116-6 (2017). 27966169 10.1007/s00216-016-0116-6

[12] Galli, R. et al. Sexing of chicken eggs by fluorescence and Raman spectroscopy through the shell membrane. *PLoS ONE* **13**(2), e0192554. 10.1371/journal.pone.0192554 (2018). 29474445 10.1371/journal.pone.0192554 PMC5824995

[13] Agri Advanced Technologies GmbH., In Ovo sex determination, Retrivied from https://www.agri-at.com/en/products/in-ovo. Accessed 21 Nov 2024 (2020).

[14] Haase, A., et al. Technische Universitaet Muenchen, Automated noninvasive determining the sex of an embryo and the fertility of a bird’s egg. U.S. Patent 11,122,778. (2021).

[15] Weissmann, A., Reitemeier, S., Hahn, A., Gottschalk, J. & Einspanier, A. Sexing domestic chicken before hatch: A new method for in ovo gender identification. *Theriogenology* **80**(3), 199–205. 10.1016/j.theriogenology.2013.04.014 (2013). 23726296 10.1016/j.theriogenology.2013.04.014

[16] Plantegg GmbH. The PLANTegg process. https://www.plantegg.de/en/ (2020).

[17] Sinclair, I., Davies, G. & Semple, H. Acoustic mist ionization mass spectrometry (AMI-MS) as a drug discovery platform. *Expert Opin. Drug Discov.* **14**(7), 609–617. 10.1080/17460441.2019.1613369 (2019). 31081699 10.1080/17460441.2019.1613369

[18] Sinclair, I. et al. Acoustic mist ionization platform for direct and contactless ultrahigh-throughput mass spectrometry analysis of liquid samples. *Anal. Chem.* **91**(6), 3790–3794. 10.1021/acs.analchem.9b00142 (2019). 30835099 10.1021/acs.analchem.9b00142

[19] Liu, C., Van Berkel, G. J., Cox, D. M. & Covey, T. R. Operational modes and speed considerations of an acoustic droplet dispenser for mass spectrometry. *Anal. Chem.* **92**(24), 15818–15826. 10.1021/acs.analchem.0c02999 (2020). 33063997 10.1021/acs.analchem.0c02999

[20] Van Berkel, G. J. & Kertesz, V. An open port sampling interface for liquid introduction atmospheric pressure ionization mass spectrometry. *Rapid Commun. Mass Spectrom.* **29**(19), 1749–1756. 10.1002/rcm.7274 (2015). 26331924 10.1002/rcm.7274

[21] Habe, T. T. et al. Ultrahigh-throughput ESI-MS: Sampling pushed to six samples per second by acoustic ejection mass spectrometry. *Anal. Chem.* **92**(18), 12242–12249. 10.1021/acs.analchem.0c01632 (2020). 32786476 10.1021/acs.analchem.0c01632

[22] Kahn, N. W., St John, J. & Quinn, T. W. Chromosome-specific intron size differences in the avian CHD gene provide an efficient method for sex identification in birds. *Auk* **115**(4), 1074–1078. 10.2307/4089527 (1998).

[23] van der Laan, T. et al. Fractionation platform for target identification using off-line directed two-dimensional chromatography, mass spectrometry and nuclear magnetic resonance. *Anal. Chim. Acta.* **1142**, 28–37. 10.1016/j.aca.2020.10.054 (2021). 33280701 10.1016/j.aca.2020.10.054

[24] MacLean, B. et al. Skyline: An open source document editor for creating and analyzing targeted proteomics experiments. *Bioinformatics* **26**(7), 966–968. 10.1093/bioinformatics/btq054 (2010). 20147306 10.1093/bioinformatics/btq054 PMC2844992

[25] Xia, J., Mandal, R., Sinelnikov, I. V., Broadhurst, D. & Wishart, D. S. MetaboAnalyst 2.0—A comprehensive server for metabolomic data analysis. *Nucleic Acids Res.* **40**, W127–W133. 10.1093/nar/gks374 (2012). 22553367 10.1093/nar/gks374 PMC3394314

[26] Drouin, N. et al. Effective mobility as a robust criterion for compound annotation and identification in metabolomics: Toward a mobility-based library. *Anal. Chim. Acta.* **1032**, 178–187. 10.1016/j.aca.2018.05.063 (2018). 30143215 10.1016/j.aca.2018.05.063

[27] Pezzatti, J. et al. A scoring approach for multi-platform acquisition in metabolomics. *J. Chromatogr. A.* **1592**, 47–54. 10.1016/j.chroma.2019.01.023 (2019). 30685186 10.1016/j.chroma.2019.01.023

[28] Koller, D., & Sahami, M. Toward optimal feature selection. In S. InfoLab (Ed.) http://ilpubs.stanford.edu:8090/208/ (1996).

[29] Steyerberg, E. W. et al. Internal validation of predictive models: Efficiency of some procedures for logistic regression analysis. *J. Clin. Epidemiol.* **54**(8), 774–781. 10.1016/s0895-4356(01)00341-9 (2001). 11470385 10.1016/s0895-4356(01)00341-9

[30] Bjornstad, S., Austdal, L. P., Roald, B., Glover, J. C. & Paulsen, R. E. Cracking the egg: Potential of the developing chicken as a model system for nonclinical safety studies of pharmaceuticals. *J. Pharmacol. Exp. Ther.* **355**(3), 386–396. 10.1124/jpet.115.227025 (2015). 26432906 10.1124/jpet.115.227025

[31] Mellor, D. J. & Diesch, T. J. Birth and hatching: Key events in the onset of awareness in the lamb and chick. *N. Z. Vet. J.* **55**(2), 51–60. 10.1080/00480169.2007.36742 (2007). 17410211 10.1080/00480169.2007.36742

[32] Selcuk, M. L. & Kayikci, F. Anatomical and embryological development of the chick cerebrum in different embryonic periods. *Vet. Med. Sci.* **11**(1), e70124. 10.1002/vms3.70124 (2025). 39792061 10.1002/vms3.70124 PMC11720722

[33] Rochat, B. Proposed confidence scale and ID score in the identification of known-unknown compounds using high resolution MS data. *J. Am. Soc. Mass. Spectrom.* **28**(4), 709–723. 10.1007/s13361-016-1556-0 (2017). 28116700 10.1007/s13361-016-1556-0

[34] Sumner, L. W. et al. Proposed minimum reporting standards for chemical analysis Chemical Analysis Working Group (CAWG) Metabolomics Standards Initiative (MSI). *Metabolomics* **3**(3), 211–221. 10.1007/s11306-007-0082-2 (2007). 24039616 10.1007/s11306-007-0082-2 PMC3772505

